# New molecular target for the phylogenetic identification of *Leptospira* species directly from clinical samples: an alternative gene to 16S rRNA

**DOI:** 10.1590/0037-8682-0333-2019

**Published:** 2020-03-16

**Authors:** Rafael Guillermo Villarreal Julio, Piedad Agudelo-Flórez, Juan Álvaro López, Ronald Guillermo Peláez Sánchez

**Affiliations:** 1 Basic Science Research Group, Graduates School - CES University. Medellín, Antioquia, Colombia.; 2 Microbiology School, Primary Immunodeficiencies Group, University of Antioquia. Medellín, Antioquia, Colombia.

**Keywords:** Leptospira, Species, Phylogenetic, Typing, rpoC

## Abstract

**INTRODUCTION:**

Phylogenetic analysis of the 16S ribosomal gene initial region is used to identify *Leptospira* isolates at the species level from clinical samples. Unfortunately, this method cannot differentiate between some intermediates and saprophytic species.

**METHODS:**

We used comparative genomic analysis between 35 *Leptospira* species to find new molecular targets for *Leptospira* species identification.

**RESULTS:**

We proposed the use of the *rpoC* gene, encoding the DNA-directed RNA polymerase β-subunit, for identifying 35 *Leptospira* species.

**CONCLUSIONS:**

The *rpoC* gene can be a molecular target to identify the main species of the *Leptospira* genus directly from clinical samples.

Leptospirosis is a globally distributed zoonotic disease caused by pathogenic bacteria of the *Leptospira* genus^1^. Currently, a phylogenetic analysis of the 16S ribosomal gene is used to identify new isolates at the species level from clinical samples^2^. Most studies use the initial gene region, 45% of which contains gene polymorphisms. Unfortunately, with this gene region, it is not possible to differentiate between *L. biflexa/L. wolbachii*, *L. meyeri/L. macculloughii/L. levettii/L. yanagawae*, *L. licerasiae/L. saintgironsiae/L. neocaledonica, L. brenneri/L. harrisiae*, and *L. venezuelensis/L. haakeii/L. hartskeerlii/L. wolffii* species^3^. Therefore, it is necessary to amplify and sequence the complete gene (1500 base pairs, approximately), which greatly decreases PCR sensitivity in the diagnosis and identification of clinical samples. Additionally, deduced phylogeny of the complete gene cannot be used to discriminate between *L. meyeri* and *L. yanagawae* ^4^. Therefore, the discovery of new molecular targets for diagnosis and species identification from clinical samples is necessary. We present a comparative genomic analysis between 35 *Leptospira* species, with the aim of finding new molecular targets for the identification of *Leptospira* species.

## Phylogenetic analysis of 16S ribosomal gene

The reference sequences of 35 *Leptospira* species were downloaded from the NCBI database. These species represent pathogenic, intermediate, and saprophytic subgroups. The evolutionary history was inferred using the Neighbor-Joining method. The tree topologies were evaluated by using a bootstrap test, and the values were obtained after 1000 replicates of the dataset. The evolutionary distances were computed using the Kimura 2-parameter method. A total of 331 positions were obtained in the final dataset. Evolutionary analyses were conducted with the Molecular Evolutionary Genetics Analysis (MEGA)-V7 software^5^.

## Orthologous proteins detection and percent protein sequence identity

The proteomes were used for the identification of orthologous proteins. The OrthoMCL (for the identification of orthologous groups in eukaryotic genomes) and OrthoVenn (a web server for genome-wide comparison and annotation of orthologous clusters across multiple species) bioinformatics tools were used to cluster proteins into orthologous groups based on the identity of their sequences^6^ ^,^ ^7^, and the InParanoid algorithm (orthologous groups with in-paralogous genes) was used to identify pairwise orthologous proteins^8^. The percentage of sequence similarity between *Leptospira* species was determined using the Rapid Annotation Using Subsystem Technology (RAS) server^9^. The percentage of sequence similarity between proteins was initially determined between 19 *Leptospira* species because the RAST web server only allowed for analyses by groups of 10 species. Subsequently, the presence of orthologous proteins was verified in the remaining 16 species. 

## Choice and analysis of candidate proteins

The proteins that were conserved among the 35 *Leptospira* species were selected for manual extraction of their coding sequences (CDS) from their genomes. These CDS were aligned using the MEGA-V7 software to identify conserved regions among CDS for the design of genus-specific primers and polymorphic regions that allow for the differentiation of species.

## Design and verification of primers

The primers were designed using the Primers3 bioinformatics program. Dimers, heterodimers, and hairpins were evaluated with the Oligo Analyzer software, and the cross-amplification test was performed with Primer-BLAST NCBI bioinformatics tool.

## Phylogenetic analysis of rpoC gene

The evolutionary history was inferred using the Neighbor-Joining method. The percentage of replicate trees in which the associated taxa clustered together in the bootstrap test (1000 replicates) was determined. The evolutionary distances were computed using the Kimura 2-parameter method. There were a total of 353 positions in the final dataset. Evolutionary analyses were conducted in MEGA-V7.

## Experimental verification of the PCR-rpoC gene

A 353 bp fragment from the *rpoC* gene was amplified by polymerase chain reaction (PCR). The reagent concentrations used for PCR standardization were as follows: primers (forward, 5´-CAAGGGGTTCATATCAACGATAA-3´; reverse, 5´-GTTCCGGCAGGGATCATGTGACC-3´), 0.4 μM; dNTPs, 0.2 mM; buffer, 1×; MgCl2, 1.5 mM; Taq polymerase, 1 unit/reaction; and DNA, 200 ng/μL. The final volume for each reaction was 25 μL. The PCR was performed in a Perkin Elmer 9700 thermocycler. The thermal cycling profile was as follows: one initial denaturation cycle at 94ºC for 5 minutes, followed by 35 cycles at 94ºC for 35 seconds, 58ºC for 45 seconds, 72ºC for 1 minute, and a final cycle at the extension temperature of 72ºC for 5 minutes.

## Leptospira detection from reference strains and clinical samples

Five pathogenic (*L. interrogans*, *L. noguchii*, *L. kirschnerii*, *L. santarosai*, and *L. borgpetersenii*), two intermediates (*L. fainei*, and *L. inadai*), and one saprophytic species (*L. biflexa*) were used for the PCR-*rpoC* standardization. Six samples from humans, rodents, and monkeys naturally infected with *Leptospira* were used to verify the usefulness of the PCR in detecting *Leptospira* from clinical samples (the samples were donated by the leptospirosis research group of the CES University). Additionally, the detection limit of PCR assay for the *rpoC* gene was evaluated using serial dilutions (1/10), starting with 100 ng of DNA from the *L. interrogans* reference strain.

## rpoC gene sequencing in humans, rodents and monkey’s samples

To confirm the species identification from the phylogenetic analysis, a 353 bp fragment of the *rpoC* gene from each sample was amplified and purified using a gel extraction kit (Qiagen). The concentration and purity were determined using a Nanodrop, whereas integrity was assessed by electrophoresis with a 1% agarose gel. All amplification products were sent to the Macrogen® company (Seoul, Korea) for sequencing. For each sample, both forward and reverse sequences were used to generate a consensus sequence using Mega7. The phylogenetic analysis of the *rpoC* gene was performed as previously described.

**FIGURE 1:**
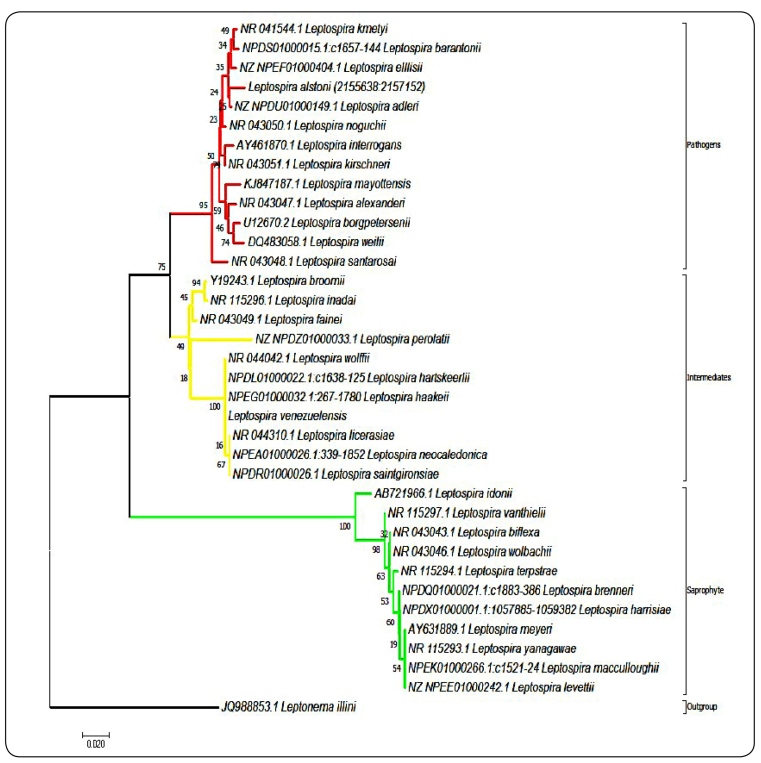
Identification of species belonging to the *Leptospira* genus using phylogenetic analysis of the 16S rRNA gene. The phylogenetic reconstructions for the 16S ribosomal gene are shown. The red, yellow, and green lines represent pathogenic, intermediate, and saprophytic species, respectively. The figure shows that the 16S ribosomal gene cannot be used to differentiate between *L. biflexa/L. wolbachii*, *L. meyeri/L. macculloughii/L. levettii/L. yanagawae*, *L. licerasiae/L. saintgironsiae/L. neocaledonica*, and *L. venezuelensis/L. haakeii/L. hartskeerlii/L. wolffii*.

Phylogenetic analysis of the partial 16S ribosomal gene resulted in the correct separation of the *Leptospira* genus from the genetically closest genus (*Leptonema illini*), an adequate separation of the pathogenic, intermediate, and saprophytic subgroups, and the ability to differentiate 20/35 species currently described^10^ with branch supports between 16% and 100%. Unfortunately, with this gene region, it was not possible to differentiate between *L. biflexa/L. wolbachii*, *L. meyeri/L. macculloughii/L. levettii/L. yanagawae*, *L. licerasiae/L. saintgironsiae/L. neocaledonica*, *L. brenneri/L. harrisiae*, *and L. venezuelensis/L. haakeii/L. hartskeerlii/L. wolffii* species (Figure 1).

Successful extraction of 35 reference genomes from the NCBI database corresponding to different *Leptospira* species was achieved (AE010300.2, AOHC00000000.2, AHOC00000000.2, AKWY00000000.2, AHMT00000000.2, GCA000332555.2, CP000348.1, CP006694.1, AHMP00000000.2, GCA_003429505.1, NZ_NPDU00000000.1, NZ_NPEF00000000.1, NZ_NPDS00000000.1, AKWX00000000.2, AHMM00000000.2, AKWZ00000000.2, AHMO00000000.2, AHOO00000000.2, GCA_002150035.1, NZ_NPDZ00000000.1, NZ_NPEA00000000.1, NZ_NPDL00000000.1, NZ_NPEG00000000.1, NZ_NPDR00000000.1, AOGY00000000.2, CP000786.1, AKXE00000000.1, AOGZ00000000.2, AOGW00000000.2, AOGX00000000.2, NZ_NPEK00000000.1, NZ_NPDQ00000000.1, NZ_NPDW00000000.1, NZ_NPDM00000000.1, NZ_RQHW00000000.1). Additionally, the reference sequences of the *rpoC* and 16S ribosomal genes were extracted manually from these genomes for the subsequent phylogenetic analyses.

A query of orthologous proteins between the 35 *Leptospira* species revealed 1650 genus-specific proteins [Supplementary data (Figure 2A)]. An amino acid identity analysis among the different species show a high genetic diversity between different *Leptospira* species. The saprophytic species were the most conserved, while the pathogenic and intermediate species showed a high genomic plasticity [Supplementary data (Figure 2B)]. Additionally, the most polymorphic proteins, but with conserved regions, were selected for primer design and subsequent phylogenetic analysis from its coding regions.

A manual evaluation of orthologous proteins conserved in the genus was made with the following inclusion parameters for the proteins: (1) the protein must be present in all species, (2) the gene must have conserved regions for genus-specific primer design, and (3) the genetic polymorphisms should allow for the differentiation of the 35 *Leptospira* species. Interestingly, high variability in nucleotide sequences and low variability in amino acid sequences were found; these findings could be related to genetic differences between the three species-subgroups and may have an important role in virulence. From these conserved proteins, we selected the DNA-directed RNA polymerase subunit beta protein (*rpoC* gene) because this protein was conserved in all species of the *Leptospira* genus, and its polymorphism permits genus, subgroups, and species identification. Additionally, this protein contains conserved regions that allow for the design of gender-specific primers. 

## Design and verification of primers

The *rpoC-F* and *rpoC-R* primers were designed in two conserved regions of the *rpoC* gene using the Primers3 bioinformatics tool. The *in silico* amplification product was 353 bp. Dimers (4.01 kcal/mol), heterodimers (4.41 kcal/mol), and hairpins (0.87 kcal/mol) were evaluated with the Oligo Analyzer software. A cross-amplification test was performed with the Primer-BLAST NCBI bioinformatics tool. No cross-amplification of sizes close to 353 bp were obtained. Only one possible amplification of 476 bp was detected in *Runella sp*, *Bacteroidetes* and *Spirosoma radiotolerans,* but the primers did not have a 100% alignment with these species; therefore, an amplification would not be obtained. 

## Phylogenetic analysis of rpoC gene

The *rpoC* gene sequences of 35 *Leptospira* species were obtained from the NCBI database. To delimit the phylogenetic analysis, the *rpoC-F* and *rpoC-R* primers were used. A common 353 bp fragment for each species was used for the phylogenetic analysis. As a result of the sequence alignment, we found that 58.07% (205 nucleotides) and 41.92% (148 nucleotides) were conserved and variable, respectively. A phylogenetic analysis of the *rpoC* gene resulted in the correct separation of the *Leptospira* genus, an adequate separation of the pathogenic, intermediate, and saprophytic subgroups, and the ability to differentiate 35 currently described *Leptospira* species with branch supports between 11% and 100% (data not shown).

## Experimental verification of the PCR-rpoC

With the PCR assay for *rpoC*, it was possible to amplify the *rpoC* gene from eight reference strains belonging to pathogenic, intermediate, and saprophytic *Leptospira* species [Supplementary data (Figure 3-A1)], and the other species were theoretically inferred. Additionally, the usefulness of the PCR assay for detecting *Leptospira* from human, rodent, and monkey samples naturally infected with *Leptospira* was verified [Supplementary data (Figure 3-A2)] with an analytical sensitivity of 10 DNA femtograms per sample [Supplementary data (Figure 3-A3)]. Furthermore, we were able to correctly identify the species in human, rodent, and monkey samples by a phylogenetic analysis of the *rpoC* gene [Supplementary data (Figure 3-B)]. The phylogenetic analysis for the *rpoC* gene correctly identified the unknown samples at the species level, and these results were 100% concordant with those obtained by the phylogenetic analysis of the 16S ribosomal gene (data not shown). Human, rodent, and monkey sequences were deposited in the NCBI database under the following codes: MK510855, MK510852, MK510856, MK510851, MK510854, MK510853, MK529909, MK529910, MK521701, MK521702, MK521699, and MK521700*.*

We present a comparative genomic analysis of 35 *Leptospira* species, finding that the *Leptospira* species shared approximately 1650 orthologous proteins, and there was a high variability in the amino acid composition of these proteins. These proteins are ideal molecular targets for the implementation of new molecular characterization tools for *Leptospira* species. In our study, we found approximately 1650 orthologous proteins that were related to vital processes of the bacteria (data not shown). A notable finding was that most of these proteins reflect the molecular speciation process of the *Leptospira* genus, which may be related to the independent evolutionary process of each subgroup. By taking advantage of this evolutionary process, we propose using the *rpoC* gene (which encodes the DNA-directed RNA polymerase subunit beta) to implement a phylogenetic identification system that will allow for the identification of unknown isolates at the species level, and to classify them according to their pathogenicity status directly from clinical samples. Additionally, the gene polymorphisms allow for the identification and differentiation of the 35 *Leptospira* species currently described via the amplification of a 353 bp fragment (amplification of a small fragment helps to improve the sensitivity of PCR assays). The *rpoC* gene fragment offers better advantages than the initial region of the 16S ribosomal gene for species identification, since it can be used to differentiate between *L. biflexa/L. wolbachii*, *L. meyeri/L. macculloughii/L. levettii/L. yanagawae*, *L. licerasiae/L. saintgironsiae/L. neocaledonica*, *L. brenneri/L. harrisiae*, and *L. venezuelensis/L. haakeii/L. hartskeerlii/L. wolffii*. 

The initial fragment of the 16S ribosomal gene can only discriminate 20 out of the 35 species currently described, while the *rpoC* gene can be used to identify all 35 species. Another advantage of the *rpoC* gene is that it can be used to differentiate between intermediate species, which may be present in environmental water sources and soils and can be sources of infection for humans and animals^11^. At the experimental level, the PCR assay for *rpoC* was successfully used to detect species belonging to pathogenic, intermediate, and saprophytic subgroups. Additionally, the PCR assay for *rpoC* with unknown samples from humans, rodents, and monkeys was successful in detecting *Leptospira* with an analytical sensitivity of 10 femtograms of DNA. Furthermore, the phylogenetic analysis for the *rpoC* gene correctly identified the unknown samples at the species level, and these results were 100% concordant with those obtained by the phylogenetic analysis of the 16S ribosomal gene (data not shown). Therefore, the PCR assay for *rpoC* could be a valuable tool for the detection and identification of *Leptospira* species directly from unknown samples with a high analytical sensitivity in addition to the absence of cross-reactions with other bacterial species and uncultivable microorganisms. Currently, the *rpoB* gene, *wzy* gene*, S10-spc-α* locus, and 23S rRNA gene are used to identify *Leptospira* species, but the 35 species of the *Leptospira* genus currently described have not been evaluated in these studies^12^ ^-^ ^15^. In conclusion, this gene could be used as a molecular target in tools to diagnose and identify the main species of the *Leptospira* genus directly from clinical samples, environmental water sources, and soils.
