# Supplementary material for: New molecular target for the phylogenetic identification of Leptospira species directly from clinical samples: an alternative gene to 16S rRNA
Source: Rev Soc Bras Med Trop. 2020 Mar 16;53:e20190333. doi: 10.1590/0037-8682-0333-2019 (PMC7094048; doi:10.1590/0037-8682-0333-2019)
Supplement: Supplementary file 1 [file 1678-9849-rsbmt-53-e20190333-suppl1.pdf]

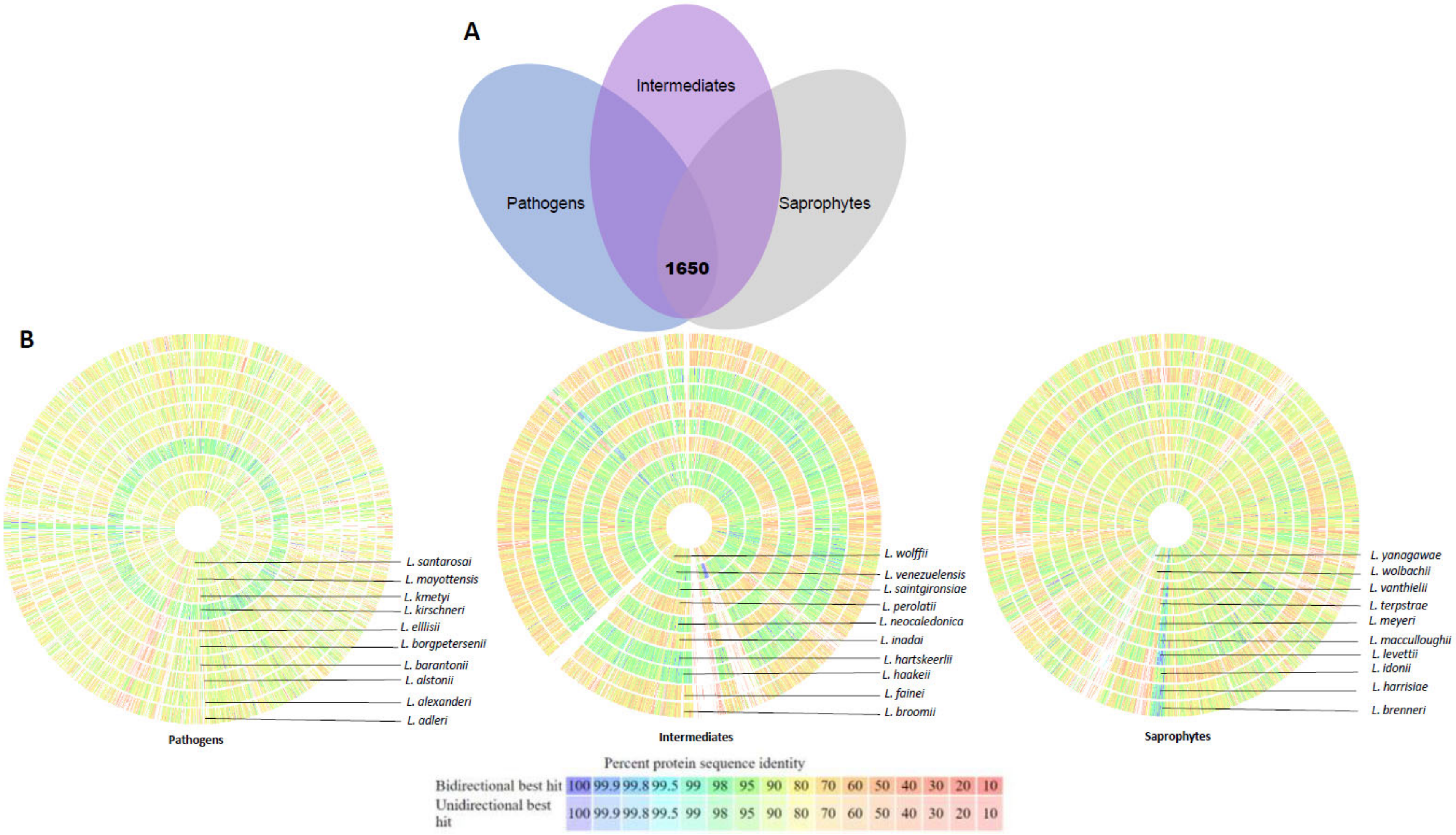

FIGURE 2: (A) Detection of orthologous proteins between 35 *Leptospira* species. The red, yellow, and green circles represent the pathogenic, intermediate, and saprophyte subgroups. The figure shows the number of orthologous proteins that are conserved in the *Leptospira* genus. The detection of orthologous proteins was performed by a combination of OrthoVenn (a web server for genome-wide comparison and annotation of orthologous clusters across multiple species), OrthoMCL (for the identification of orthologous groups for eukaryotic genomes) and InParanoid (orthologous groups with in-paralogous) bioinformatics tools. **(B)** The figure shows the percentages of similarities between proteins sequences belonging to pathogenic, intermediate, and saprophytic *Leptospira* subgroups. The detection of orthologous proteins and comparison between the genomes were carried out using the Rapid Annotation Using Subsystem Technology (RAST) server. The percentages of similarities between protein sequences of the genomes is shown on a color scale (between 10% and 100%) and a bidirectional amino acid reading methodology.
