# Supplementary material for: New molecular target for the phylogenetic identification of Leptospira species directly from clinical samples: an alternative gene to 16S rRNA
Source: Rev Soc Bras Med Trop. 2020 Mar 16;53:e20190333. doi: 10.1590/0037-8682-0333-2019 (PMC7094048; doi:10.1590/0037-8682-0333-2019)
Supplement: Supplementary file 2 [file 1678-9849-rsbmt-53-e20190333-suppl2.pdf]

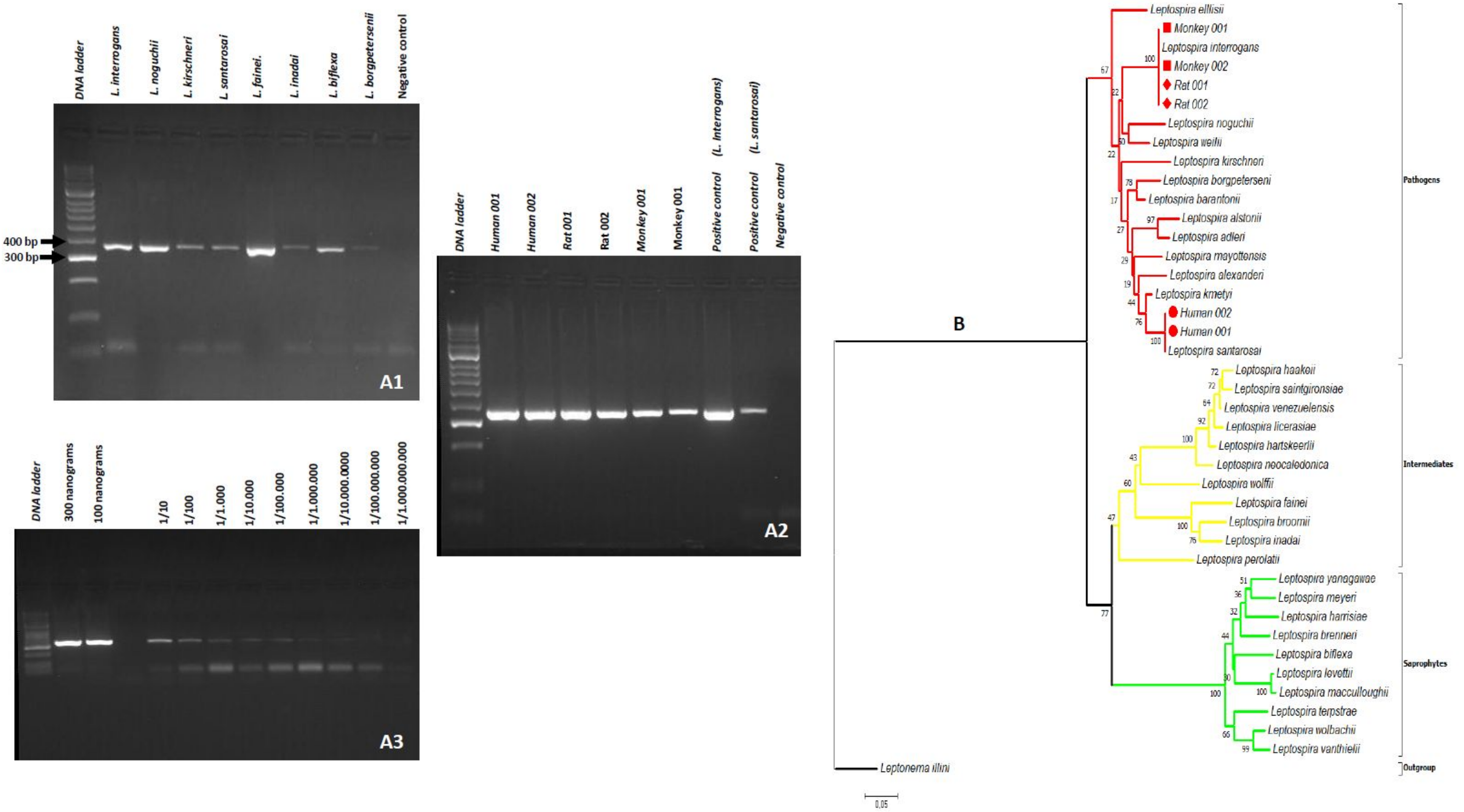

FIGURE 3: (A) PCR standardization of the *rpoC* gene (which encodes the DNA-directed RNA polymerase subunit beta). (**A1**) The amplification of the *rpoC* gene in five pathogenic, two intermediate, and one saprophyte species. (**A2**) The detection of *Leptospira* in human, rodent, and monkey samples. (**A3**) The detection limit or analytical sensitivity of the PCR using the *rpoC* gene as molecular target, achieving an amplification of up to 10 femtograms of DNA. **(B)** *Leptospira* isolates that were identified at the species level. The figure shows the identification at the species level in human, rodent, and monkey samples, which were naturally infected with *Leptospira,* by a phylogenetic analysis of the *rpoC* gene. The circles, diamonds, and squares represent the human, rodent, and monkey isolates, respectively.
